# Supplementary material for: Development of a sequencing system for spatial decoding of DNA barcode molecules at single-molecule resolution
Source: Commun Biol. 2020 Dec 18;3:788. doi: 10.1038/s42003-020-01499-8 (PMC7749132; doi:10.1038/s42003-020-01499-8)
Supplement: Supplementary file 2 — Supplementary Information [file 42003_2020_1499_MOESM2_ESM.pdf]

Supplementary Information

Development of a sequencing system for spatial decoding of DNA barcode molecules at single-molecule resolution

Yusuke Oguchi[1,2,3*], Hirofumi Shintaku[2], Sotaro Uemura[1*]

[1]Department of Biological Sciences, Graduate School of Science, The University of Tokyo, 7-3-1 Hongo, Bunkyo, Tokyo, 113-0033, Japan

[2]RIKEN Cluster for Pioneering Research, 2-1 Hirosawa, Wako, Saitama, 351-0198, Japan

[3]JST, PRESTO, 4-1-8 Honcho, Kawaguchi, Saitama, 332-0012, Japan

[*]To whom correspondence should be addressed. Email: yusuke.oguchi@riken.jp, uemura@bs.s.u-tokyo.ac.jp

Supplementary Figures 1 – 13

Supplementary Tables 1 – 2

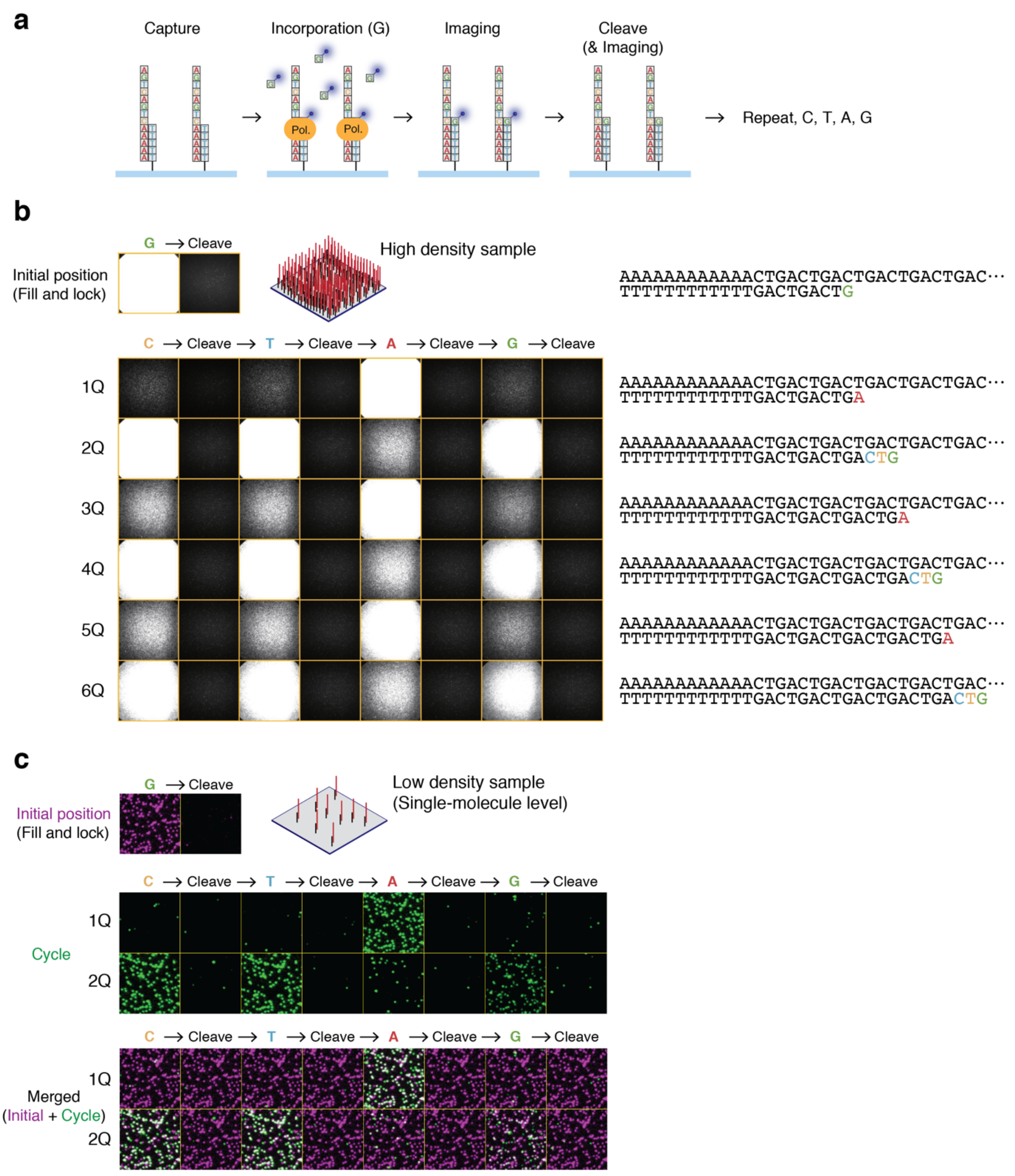

Supplementary Fig. 1: Validation of virtual terminator incorporations on the smSBS system with a simple sequence sample. a) Schematic illustration of smSBS procedures. 1) The sample used here had a brief sequence of 5′-$(AGTC)_8A_{12}$ and was captured by 5′-$T_{12}(GACT)_2$ bound to the flow cell surface. Note that the illustration does not exactly illustrate hybridization, as the repeating sequences in the captured oligo $(GACT)_2$ were omitted. 2) First, virtual terminators (VT) were incorporated with a sequencing polymerase. 3) After washing non-incorporated VTs, fluorescence images were captured. 4) Fluorophores and blocking groups bound to VTs were cleaved, and fluorescence images were again captured to confirm cleavage. **b**) Fluorescence images showing VT incorporations and cleaving

fluorophores with a high capture density sample achieved with 1 nM of 5′-$(AGTC)_8A_{12}$. As shown in (a) the first VT incorporation (in this case, G) image was indicated as the "initial position" and subsequent sequence cycles were repeated in the order of C, T, A, and G. White color in each image indicates the signal from fluorophores bound to VTs. At the 1st quad, only VT-A should be incorporated into the sample, and subsequently, at the 2nd quad C, T, and G should be incorporated. In fact, these expected incorporations were observed correctly as a white image. Note that, for example, "A" of the 2nd quad also showed slight incorporations with lower intensity compared to that with the expected incorporations. We attributed this to the phase shift incorporations of the reads that did not incorporate A at the 1st quad. However, nearly all molecules were incorporated simultaneously as expected at least until 6Q as seen here. **c**) The same example is shown in (b) however, with a low capture density sample. Next 25 pM of the sample was captured on the flow cell. In this observation, individual molecules were observed as single spots. The first position spots (colored in magenta) and the images obtained during sequencing cycles (colored in green) were merged, indicating the VT incorporations occurred at the same position as the initial position. Before merging these images, stage drift was corrected.

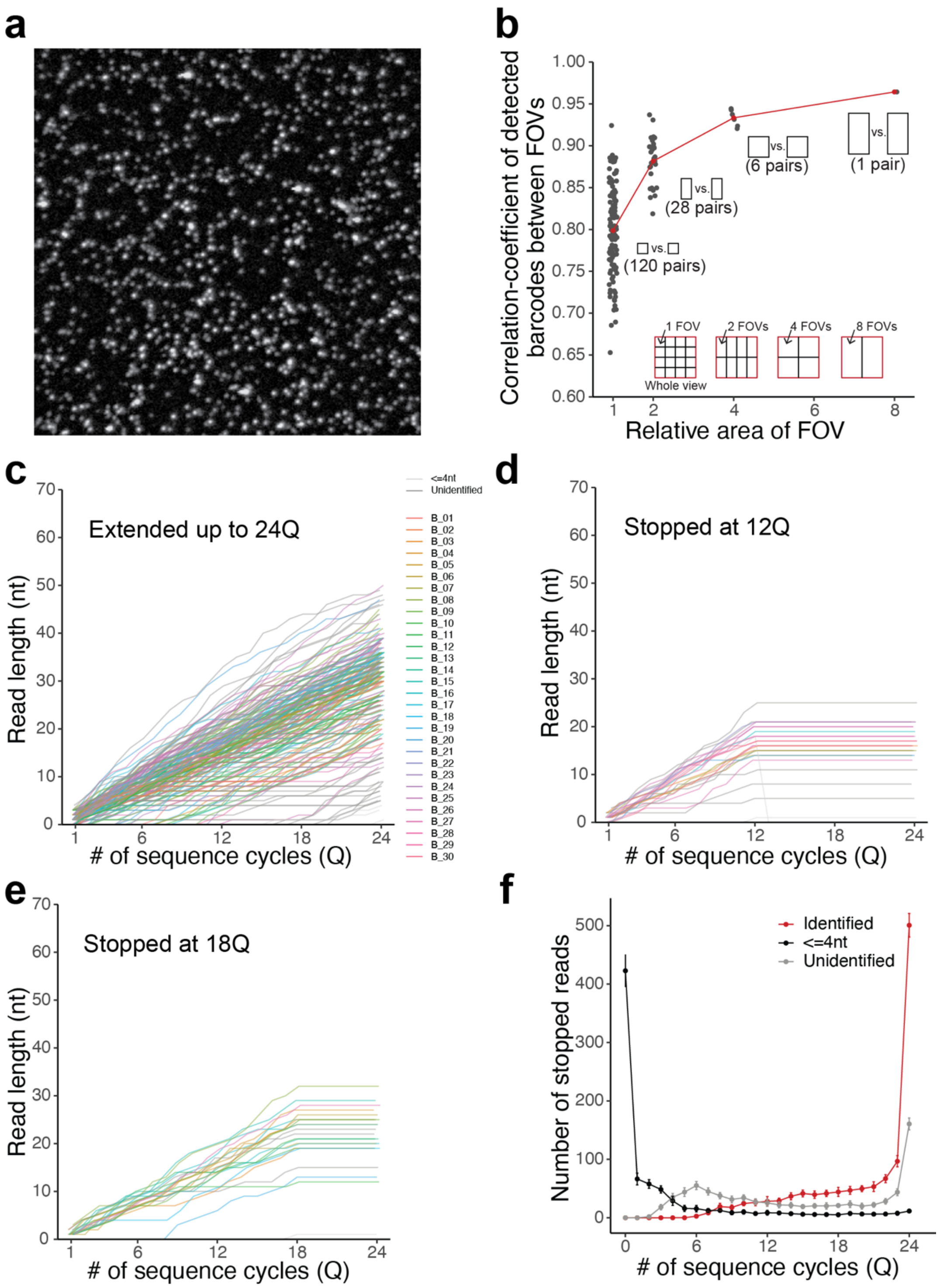

Supplementary Fig. 2: Additional results for Fig.1. a) Raw fluorescence image shown in Fig. 1d. **b**) Bias-free barcode decoding between scanning FOV. Cross-correlation of detected barcode counts was calculated by changing the area of FOVs as shown in the inset. **c-e**) Typical examples of virtual terminator incorporations to individual molecules shown in Fig. 1f are re-plotted according to the following three categories, **c**) extend up to the last quad (24Q), **d**) stopped at 12Q, and **e**) stopped at 18Q. **f**) Frequency of quads that terminated extensions are shown by identified reads, unidentified reads and reads less than 5 nt.

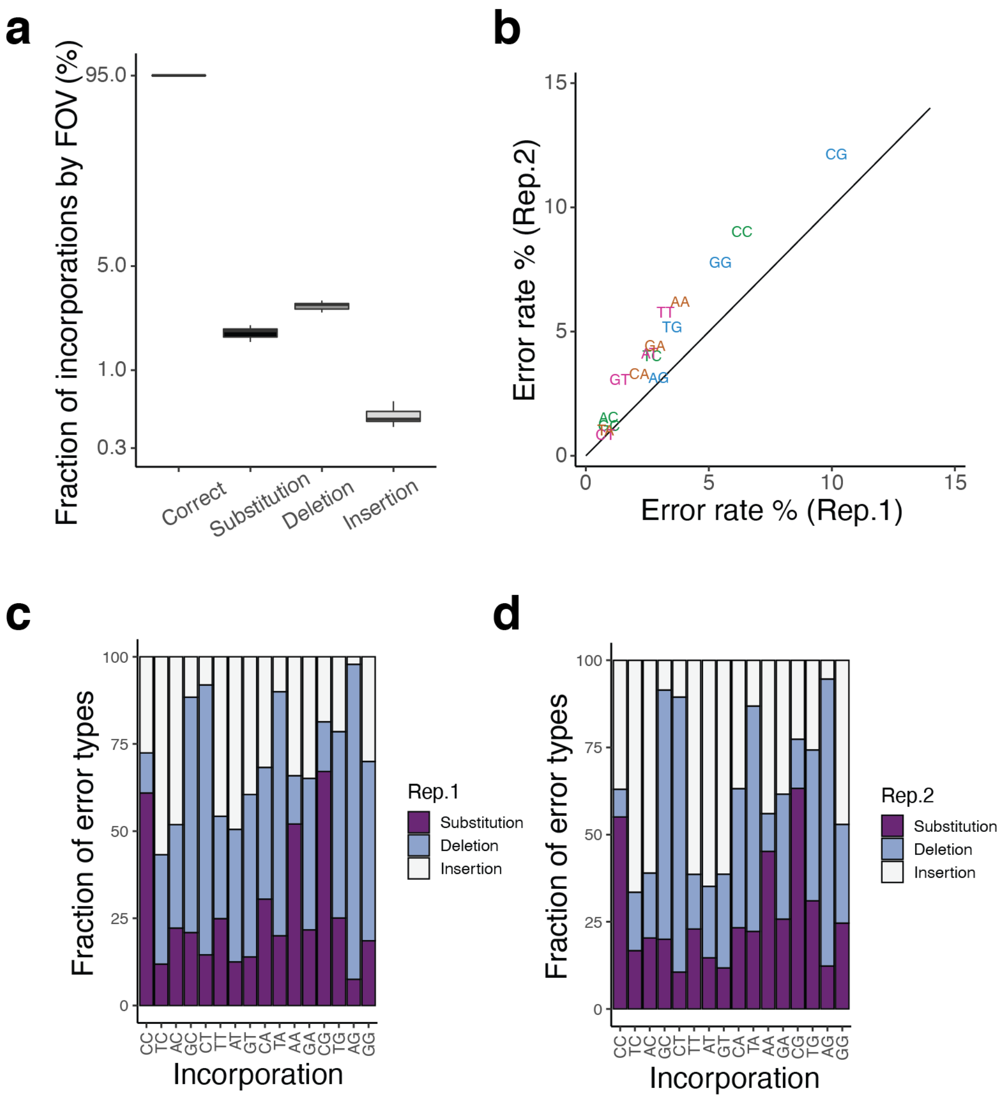

Supplementary Fig. 3: Error rate analysis. a) Accuracy of VT incorporation. These values were calculated by each FOV and averaged. Error bars show standard deviation. **b-d)** Error rate of the second incorporation for two subsequent incorporations. **b**) Comparison in the error rate of the second incorporation between technical replicates (rep.1 vs. rep.2). For instance, CG indicates the error rate of G incorporation after the C incorporation. Fraction of error types by each subsequent incorporation for technical replicates 1 (**c**) and 2 (**d**).

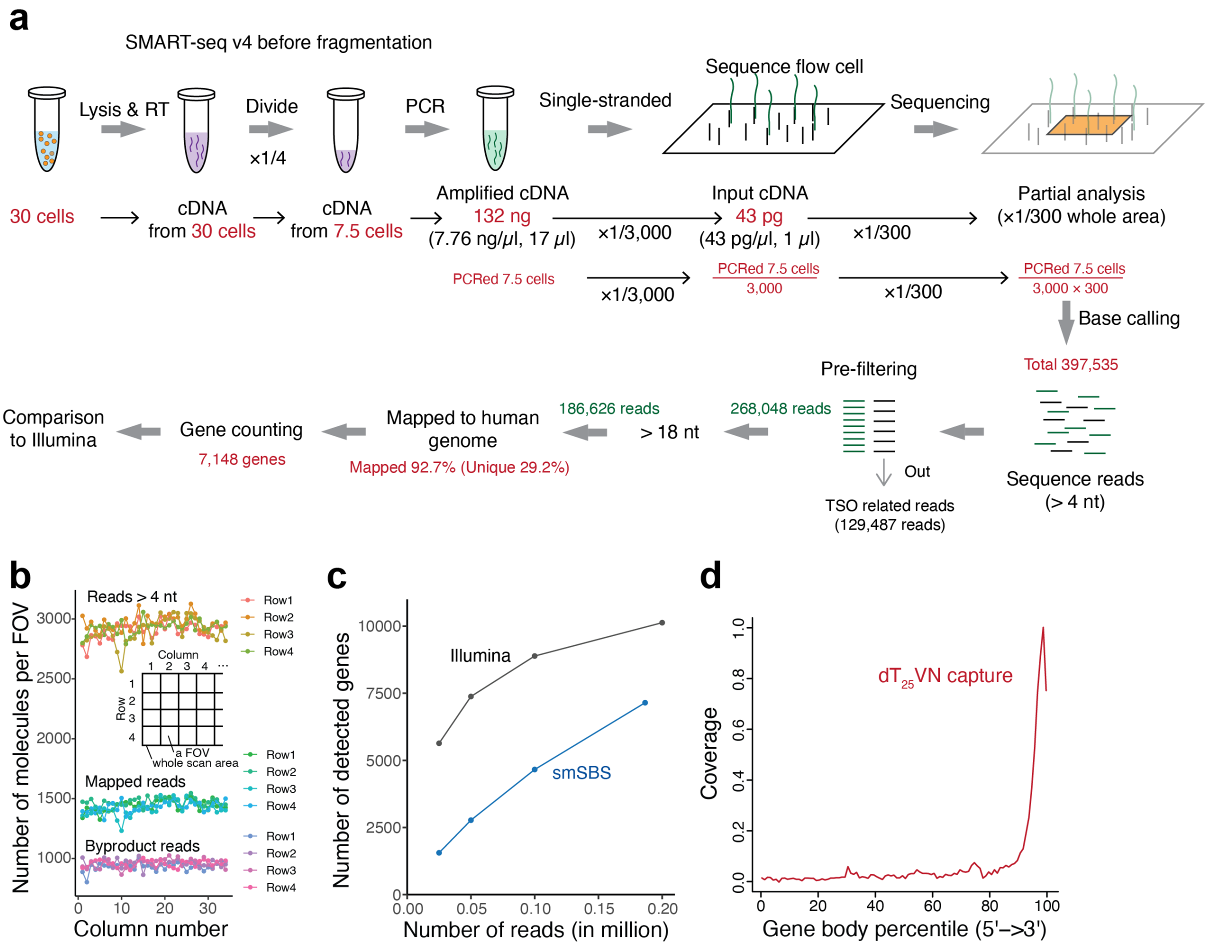

Supplementary Fig. 4: Application of smSBS to a small quantity sample of ~10 pg cDNA library of K562 cells. a) The entire experimental procedure shown in Fig. 2. **b**) Number of observed molecules per FOV. **c**) Down-sampling analysis of the number of detected genes. For Illumina data, cDNA library prepared from a 200 pg of total RNA extracted from bulk whole cells was measured. We assumed that the RNA amount extracted from ~10 cells was comparable to that from 200 pg of total RNA extracted from bulk whole cells. **d**) Gene-body coverage of smSBS with $dT_{25}VN$ capture oligo instead of TSO capture oligo.

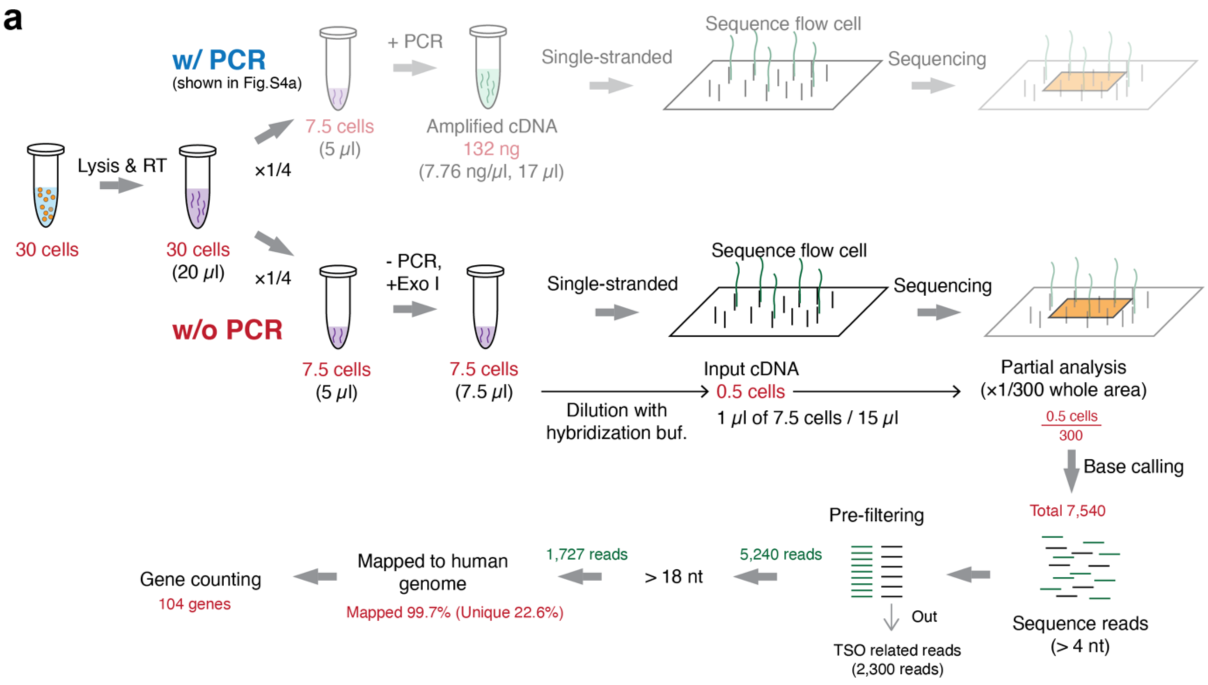

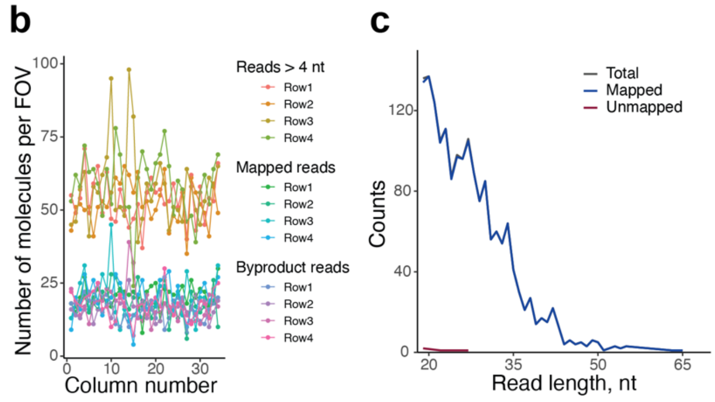

d

			+PCR	-PCR
Number of reads		Total reads (> 4 nt)	397,535	7,540
	Byproducts	TSO	85,478	975
		Comp_TSO	6,052	1,259
		T30	4,204	6
		A30	28,993	27
		(AT)15	4,760	33
		> 4 nt - Byproducts	268,048	5,240
	Mapped	> 18 nt	186,626	1,727
		Total mapped	173,051	1,721
		Uniquely mapped	54,581	460
		Multimapped	118,470	1,261
		Unmapped	13,575	6
Detected genes			7,148	104

Supplementary Fig. 5: Measurement of amplification-free cDNA comparable amount to that from a single cell. a) Scheme for measurement of an amplification-free sample. Here we used the same cDNA molecules synthesized for the test shown in Supplementary Fig. 4, however, without amplification. Following cDNA synthesis with 30 cells, the sample was divided into four aliquots. One of the four was further treated with exonuclease I to remove free RT and TSO primers and sequenced without PCR amplification. **b**) Number of detected molecules per FOV. **c**) Read length distribution following 24Q sequencing; reads less than 19 nt and containing byproducts were filtered out. **d**) Comparison in the number of reads obtained with (shown in Supplementary Fig. 4) and without PCR.

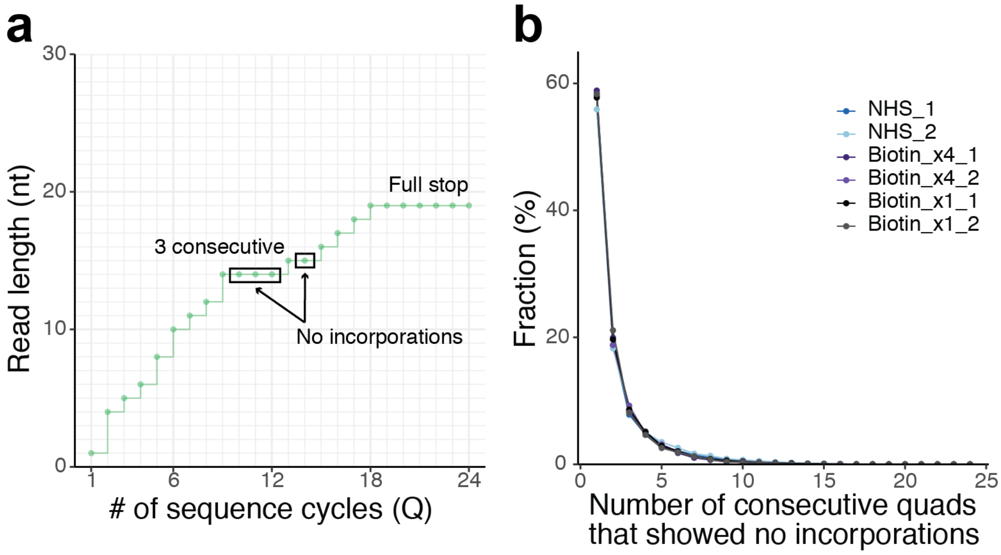

Supplementary Fig. 6: Additional data for the impact of binding strength between capture oligos and the flow cell to sequencing performance. a) A typical incorporation trace showing temporal stops. Incorporation of virtual terminators must ideally be observed at least once by each quad (since each quad has a chance to incorporate all four nucleotides (CTAG order)). However, at certain quads, no incorporation was observed (temporal stop of the extension). Further, quads showing no incorporation occurred consecutively (in this trace, one event of three consecutive stops was observed). **b**) Fraction of the number of consecutive stops. Definition of a consecutive stop is shown in a. No differences were observed between all three conditions.

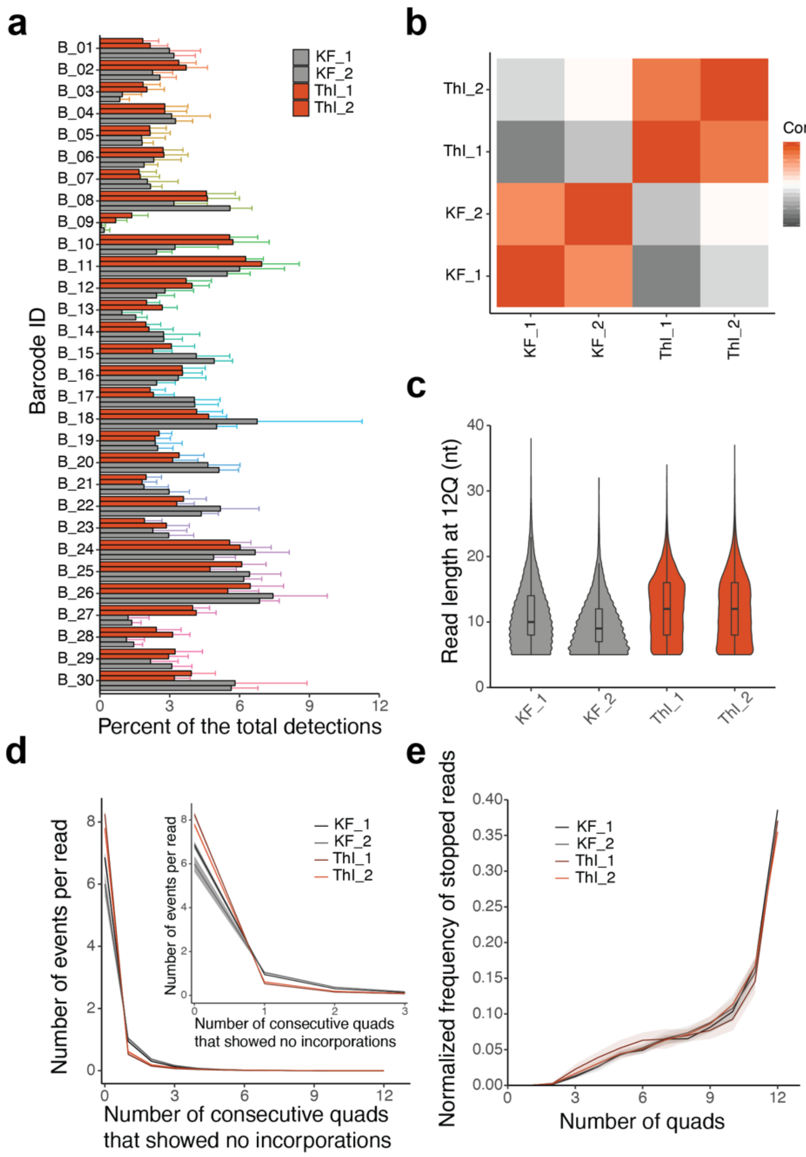

Supplementary Fig. 7: Comparison in the sequencing performance between ThI polymerase and the original polymerase of smSBS, KF. a) Detection of 30 types of DNA barcodes in the same manner as shown in Fig.1b but comparing different polymerases of Klenow Fragment exo- (KF) and Therminator I (ThI), shown in grey and red, respectively. Note, we conducted 12Q sequencing for this test. All conditions were repeated twice. **b**) Cross-correlation of the relative counts (percent of the total detected counts) between conditions shown in a. **c**) Read length distributions of KF (grey) and ThI (red); reads less than 5 nt were filtered out. Average read lengths of ThI were longer than those of KF (the difference was statistically significant with $p < 2.2 \times 10^{-16}$). **d**) Fraction of the number of consecutive stops. The frequency of consecutive stops observed in individual FOVs was normalized by the number of reads detected in the FOV. Colored ribbon plots represent SD. **e**) Normalized frequency of stopped (full stop) reads at each quad. The number of full stop events was counted and normalized by individual FOVs. Note, at 12Q, the value indicates the fraction of reads that reached 12Q. Colored ribbon plots represent SD.

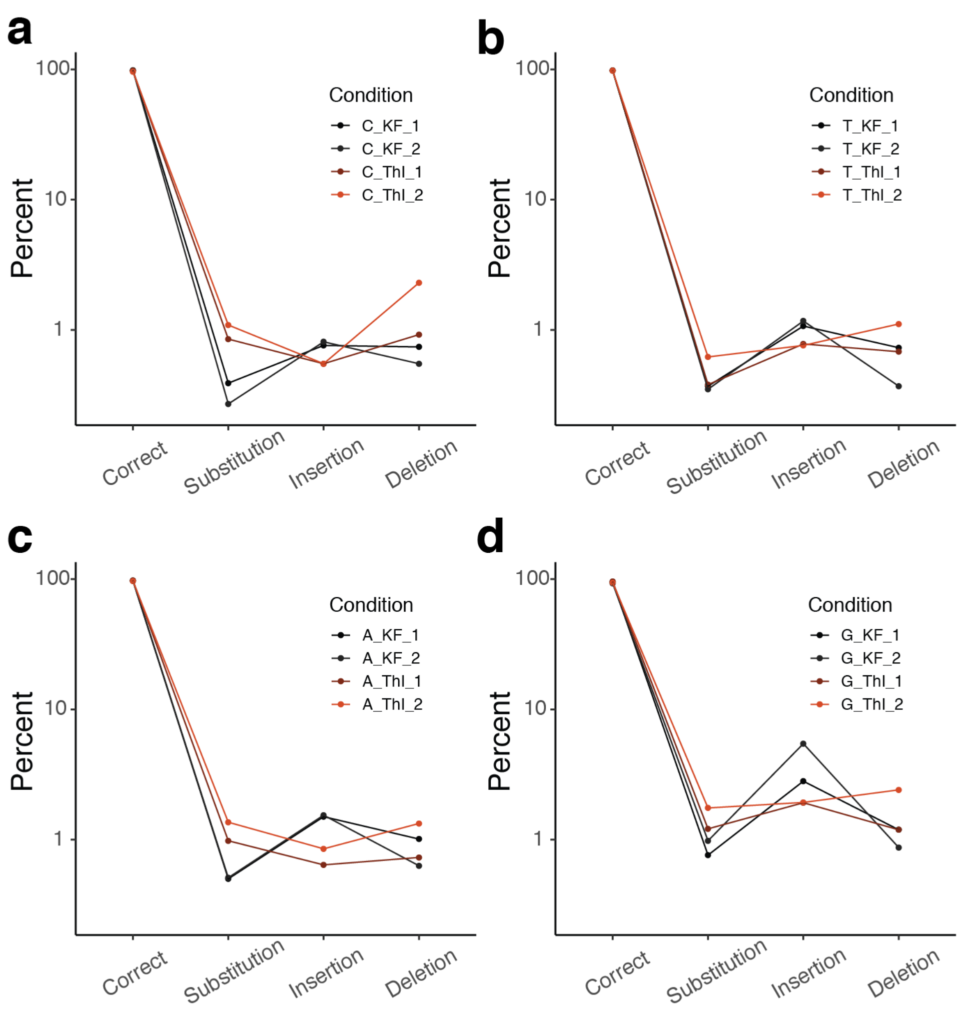

Supplementary Fig. 8: Comparison of incorporation accuracy between ThI and KF. The incorporation accuracy of C, T, A, and G are shown in **a**), **b**), **c**), and **d**), respectively. The x-axis shows incorporation type, 'correct' denotes the correct incorporation, while the other three labels represent incorrect incorporations corresponding to substitution error, insertion error, and deletion error.

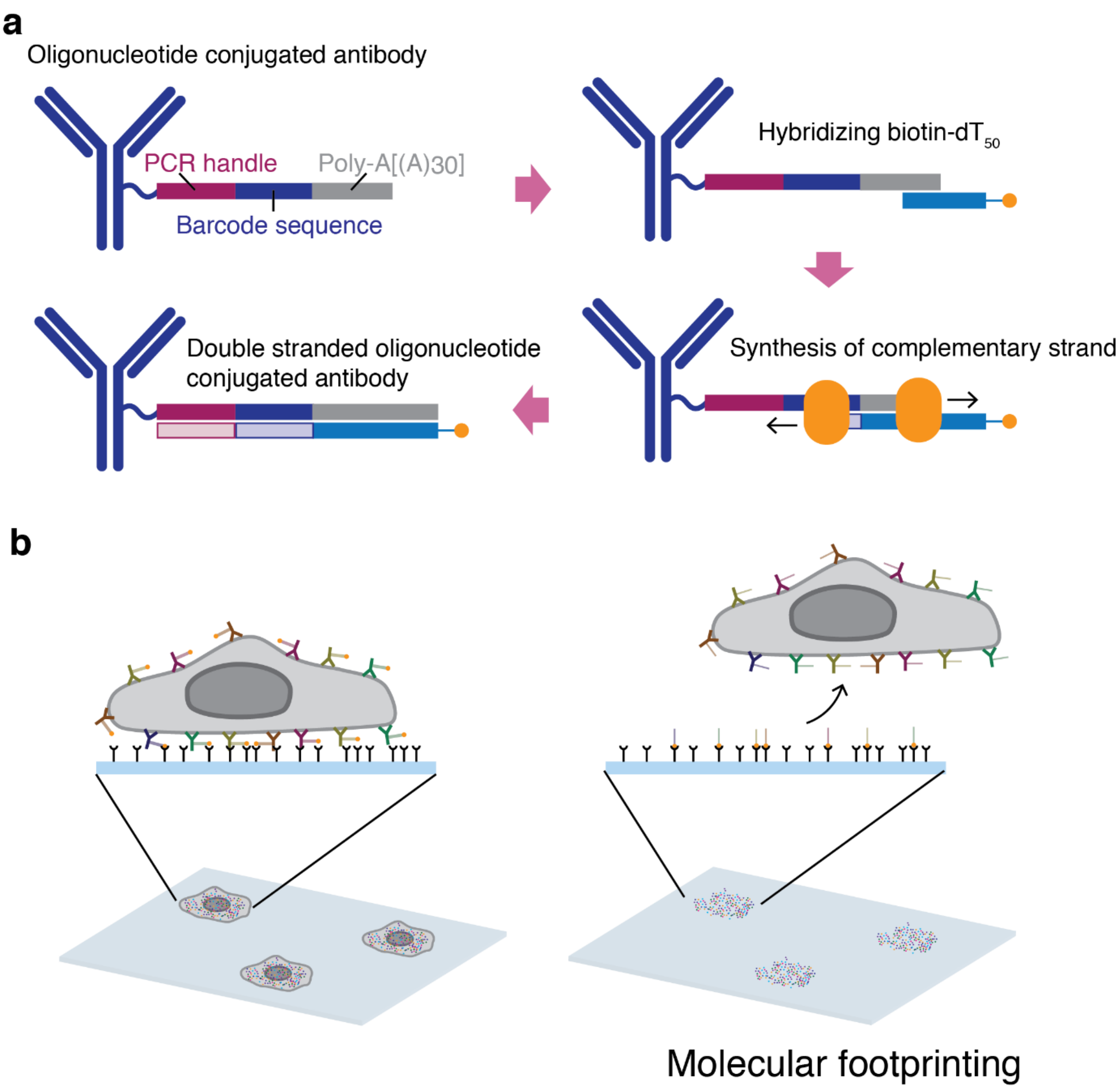

Supplementary Fig. 9: Preparation of biotinylated and double-stranded Ab-Oligos and their novel application to detection of cell surface marker. a) Oligonucleotide conjugated antibodies (Ab-Oligo) used in this study are commercially available. A single-molecule of Ab-Oligo having one or two molecules of the oligonucleotide corresponding to the antibody (1), were hybridized with biotin×1-dT_{50} or biotin×4-dT_{50} (2). The complementary strand was synthesized with a polymerase (3 to 4). **b**) A possible application utilizing biotinylated and double-stranded Ab-Oligo (Ab-dsOligo). The cells labeled with Ab-dsOligo molecules are captured on the sequencing flow cell via biotin-avidin interactions. Subsequently, as shown in Fig.4a, the cells are dissociated (washed away) from the sequencing flow cell, so that the complementary strand of Ab-Oligos is left on the surface, maintaining the distribution of the molecule on a single-cell. By sequencing these molecules as shown in Fig.4, we will be able to obtain their spatial information at the single-molecule level.

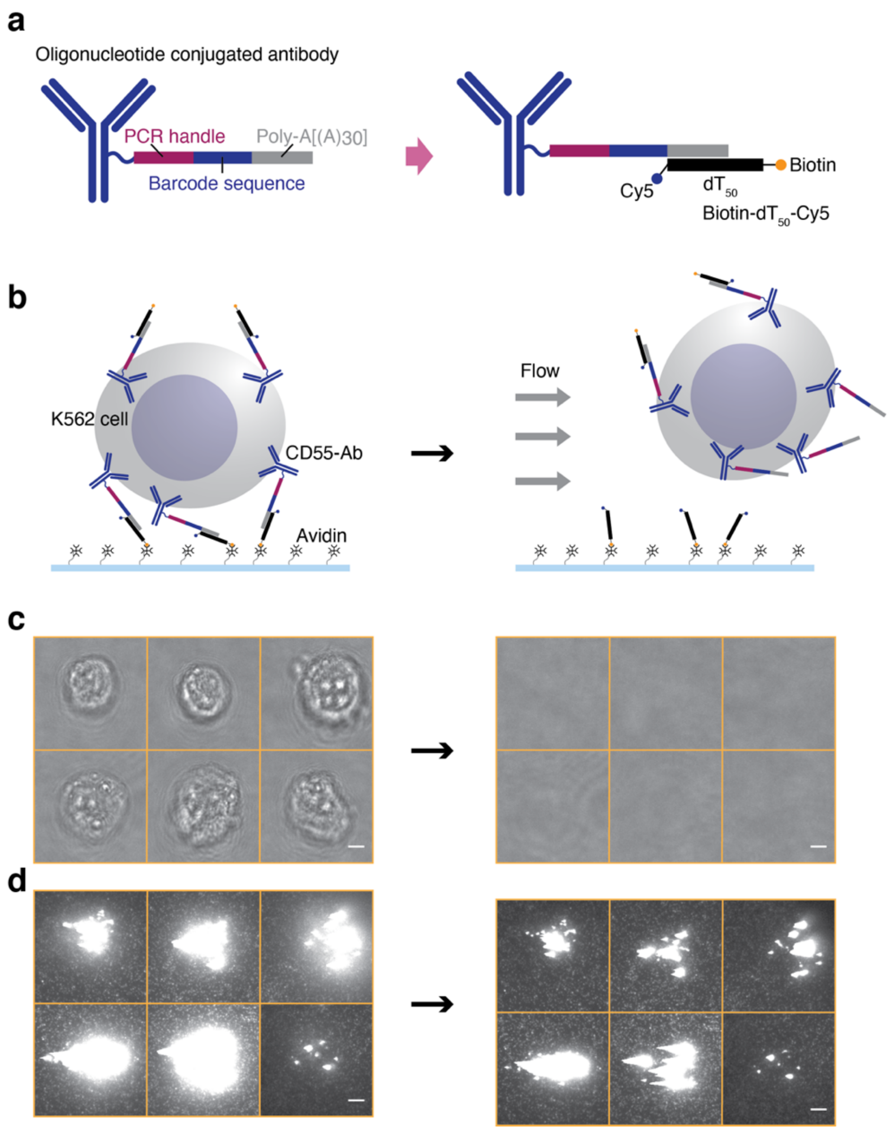

Supplementary Fig. 10: A proof-of-concept experiment for molecular foot-printing with K562 cells. a) Instead of the complementary sequence shown Fig. 5a, here, the 5′-Biotin-dT$_{50}$- Cy5-3′ probe was hybridized without a further polymerase extension reaction. **b**) Schematic illustration of the experiment with K562 cells. K562 cells bound Ab::epitope were introduced into a flow cell and captured onto the surface via biotin-avidin interactions (left panel). After observing the K562 cells capturing on the surface, the K562 cells were washed from the flow cell via a pressure driven flow, while the complementary sequences remained on the surface and were easily visualized by Cy5 molecules (right). **c**) Bright field images corresponding to the schematic shown in **b**. **d**) Fluorescence (TIRF) images corresponding to the schematic shown in **b**. After confirming the K562 cells were washed away from the original position, the same FOV was observed. Scale bars shown in c and d indicate 5 μm.

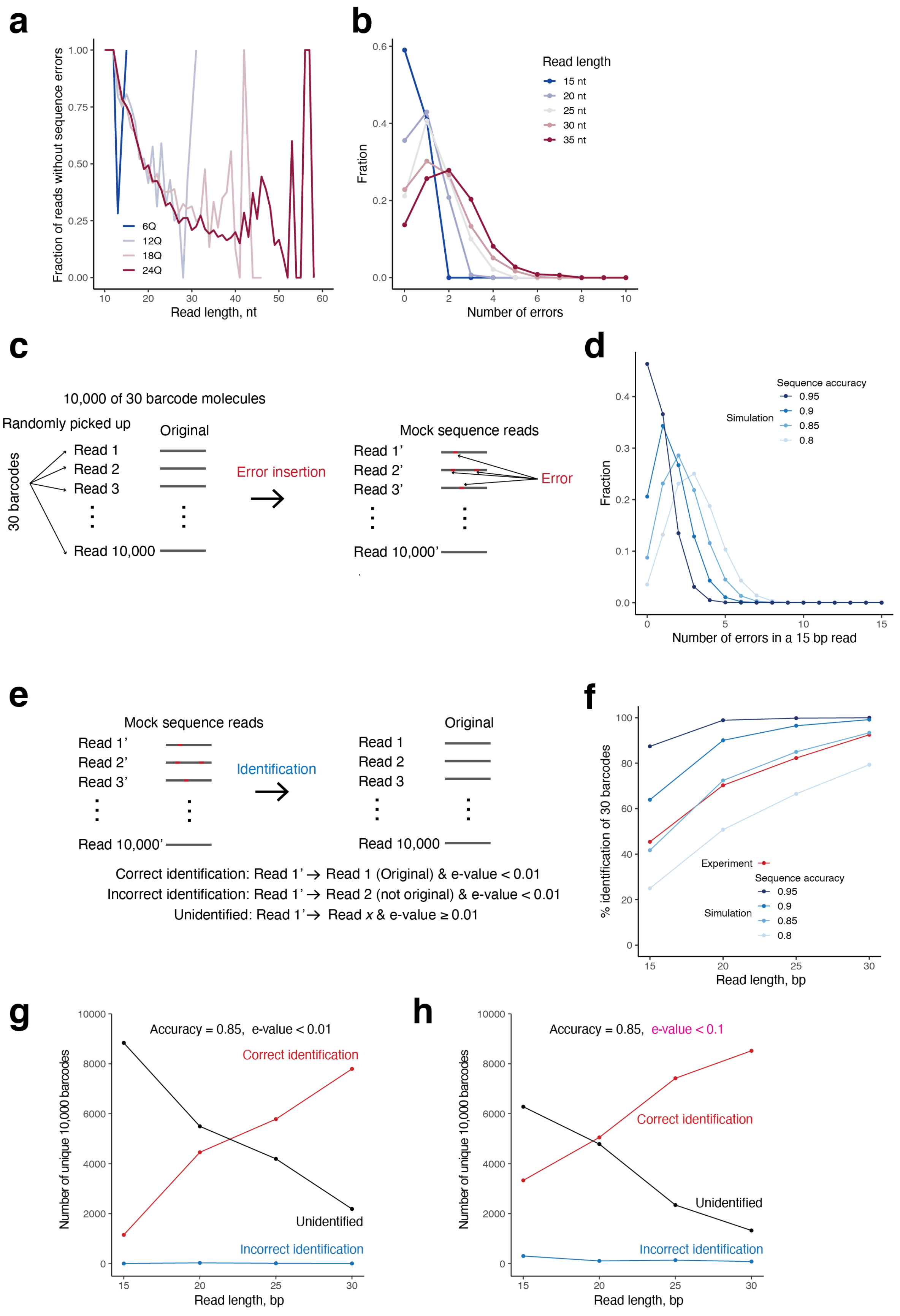

Supplementary Fig. 11: Model analysis estimating the identification efficiency of barcode molecules. (**a, b**) The number of errors in a sequence read was obtained experimentally. The fraction

of error containing reads (having at least one error) increased with the increase in read length (**a**). Distribution of the number of errors in a read for 24Q (**b**). (**c-f**) Validation of the model analysis with the experimental identification of 30 barcode molecules. **c**) Generation of mock sequence reads for this simulation. First, 10,000 barcode molecules were randomly sampled from the 30 barcode molecules with replacement. Next, we assumed that the sequencing reads (each base, as per the Bernoulli trials) would mimic the error-prone experimental process of sequencing (**d**). The fraction of reads containing n errors ($P(n)$) should be governed by a binomial distribution ($P(n) = {}_{N}C_{n}(1\text{-}p)^{n}p^{(N-n)}$, where p: sequencing accuracy, N: read length, n: number of errors per read). Based on the distribution, we created mock sequencing reads by inserting errors (here, we simply inserted substitution errors) into the original sequences. **e**) Identification of mock sequencing reads by aligning them to the original 30 barcode sequences with BLAST. **f**) Comparison of simulated identification efficiencies with the experimentally obtained result. The result of simulation with $p = 0.85$ provides a suitable fit for the experiment. (**g, h**) Estimation of the identification efficiency of 10,000 types of unique molecules. Briefly, we estimated an identification efficiency of 10,000 types of unique molecules with read lengths ranging from 15 to 30 bp, with $p = 0.85$ (**g**). First, we sampled 10,000 molecules from a uniform distribution of DNA barcodes 30 nt in length (~4^{30} possibilities) without replacement, followed by error insertion to create mock sequencing reads. The mock sequencing reads of the 10,000 types of unique molecules were identified via alignment with the original sequences using BLAST. The analysis with an e-value < 0.01 shows almost no incorrect identifications; therefore, a looser threshold, such as an e-value < 0.1, can be applied, resulting in an increased identification efficiency, especially for 15 bp read length sequences (**h**). The process of identification of molecules is described in e.

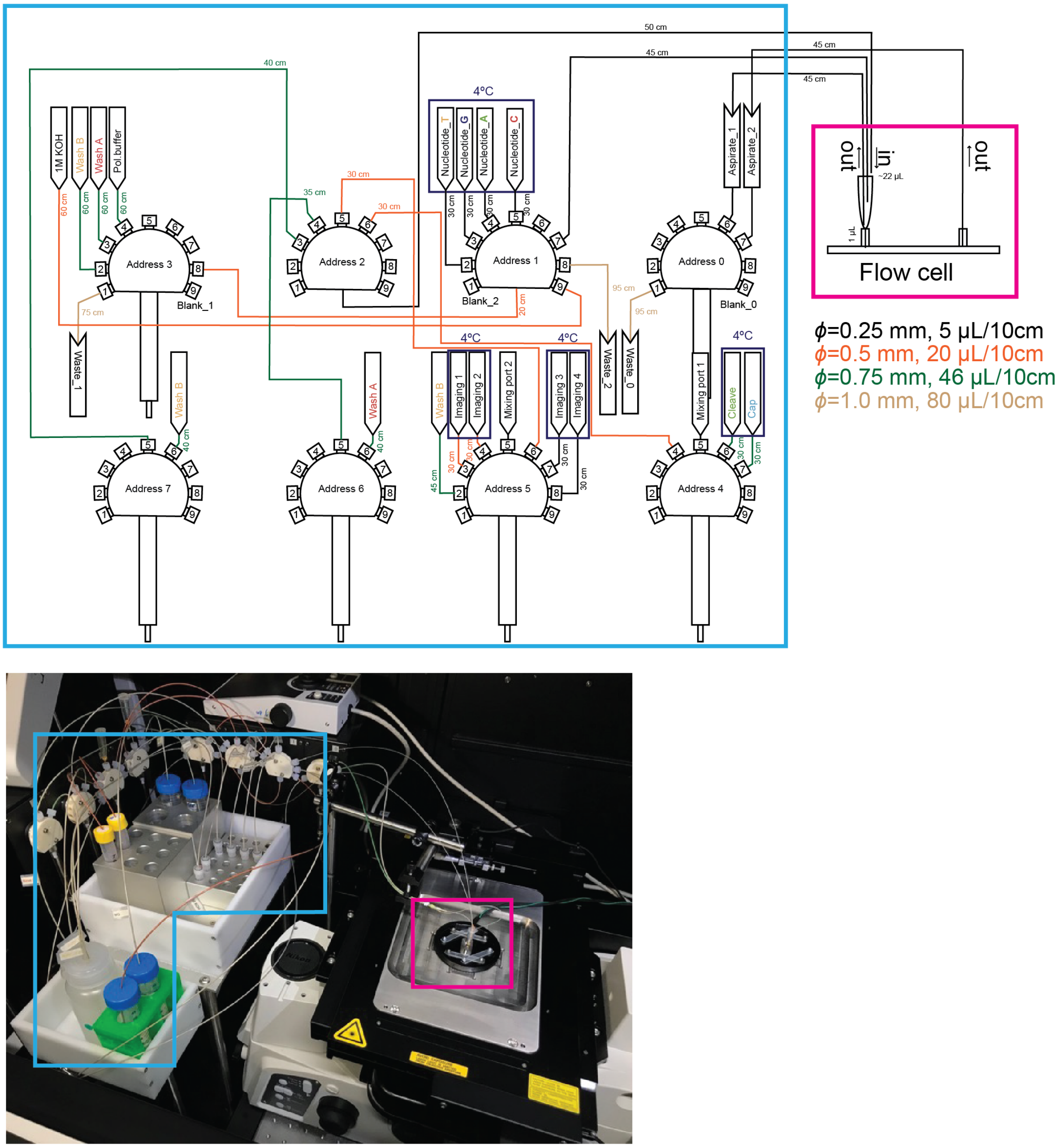

Supplementary Fig. 12: Detailed information for the fluidics system of the smSBS. A photo of our system shown in the bottom, also shown in Fig.1a, is highlighted by rectangles corresponding to those in the fluidics diagram shown above.

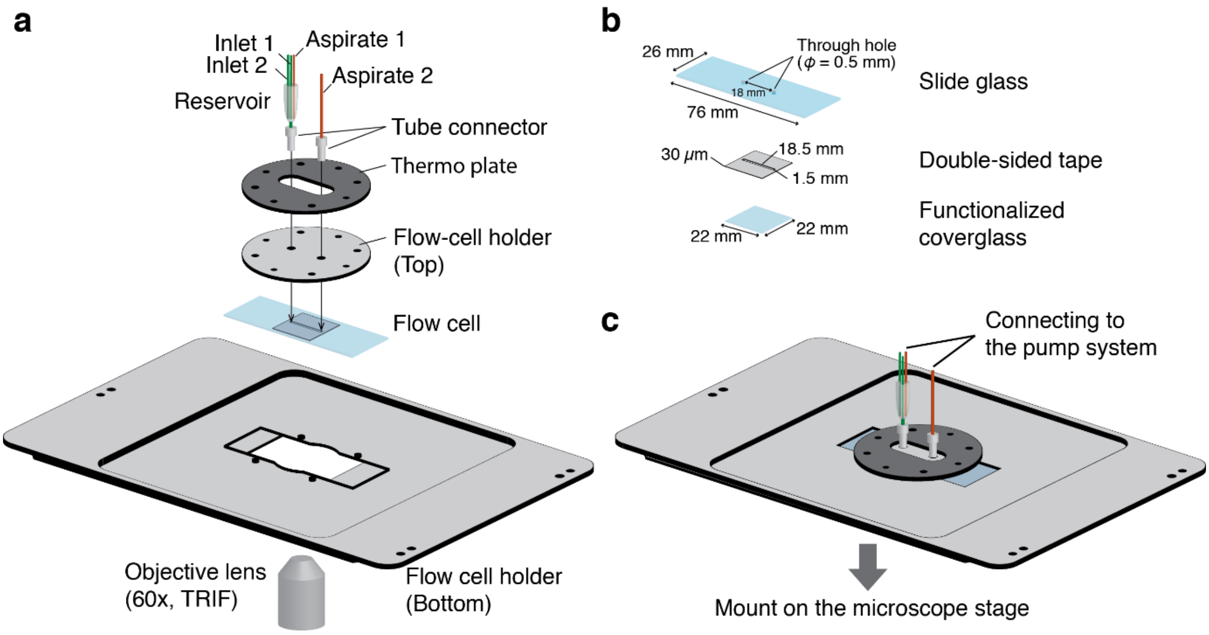

Supplementary Fig. 13: Design of the flow cell and its holder with which to mount the microscope stage and connect to the fluidic system. a) An expanded view of our system around the flow cell. **b**) An explorative view of our flow cell. **c**) 3D view of assembled components shown in a.

Supplementary Table 1: DNA barcode oligonucleotide sequences.

Barcode ID	Sequence (5′–3′)
B_01	AATCTCCAAAAAAAAGGCTCCAAAAGGAGCCTTTAATTGTATCGGTTTATCAGCTT
B_02	AATTGCGAATAATAATTTTTTCACGTTGAAAATCTCCAAAAAAAAGGCTCCAAAAG
B_03	GGAGTGAGAATAGAAAGGAACAACTAAAGGAATTGCGAATAATAATTTTTTCACGT
B_04	TTTTGCTAAACAACTTTCAACAGTTTCAGCGGAGTGAGAATAGAAAGGAACAACTA
B_05	GACGTTAGTAAATGAATTTTCTGTATGGGATTTTGCTAAACAACTTTCAACAGTTT
B_06	GTAACGATCTAAAGTTTTGTCGTCTTTCCAGACGTTAGTAAATGAATTTTCTGTAT
B_07	AGCATTCCACAGACAGCCCTCATAGTTAGCGTAACGATCTAAAGTTTTGTCGTCTT
B_08	TTCGTCACCAGTACAAACTACAACGCCTGTAGCATTCCACAGACAGCCCTCATAGT
B_09	TGATAATCAGAAAAGCCCCAAAAACAGGAAGATTGTATAAGCAAATATTTAAATTG
B_10	AGGAAGATTGTATAAGCAAATATTTAAATTGTAAACGTTAATATTTTGTTAAAATT
B_11	AAATTGTAAACGTTAATATTTTGTTAAAATTCGCATTAAATTTTTGTTAAATCAGC
B_12	TGCAAATCCAATCGCAAGACAAAGAACGCGAGAAAACTTTTTCAAATATATTTTAG
B_13	GCCTGTTTATCAACAATAGATAAGTCCTGAACAAGAAAAATAATATCCCATCCTAA
B_14	TAGATGGGCGCATCGTAACCGTGCATCTGCCAGTTTGAGGGGACGACGACAGTATC
B_15	GATCCCCGGGTACCGAGCTCGAATTCGTAATCATGGTCATAGCTGTTTCCTGTGTG
B_16	TACCTACATTTTGACGCTCAATCGTCTGAAATGGATTATTTACATTGGCAGATTCA
B_17	TAAAGCTAAATCGGTTGTACCAAAAACATTATGACCCTGTAATACTTTTGCGGGAG
B_18	TTAGACAGGAACGGTACGCCAGAATCCTGAGAAGTGTTTTTATAATCAGTGAGGCC
B_19	ACCGCCTGGCCCTGAGAGAGTTGCAGCAAGCGGTCCACGCTGGTTTGCCCCAGCAG
B_20	GACCAACTTTGAAAGAGGACAGATGAACGGTGTACAGACCAGGCGCATAGGCTGGC
B_21	ACCTTGCTTCTGTAAATCGTCGCTATTAATTAATTTTCCCTTAGAATCCTTGAAAA
B_22	CAGGCGGATAAGTGCCGTCGAGAGGGTTGATATAAGTATAGCCCGGAATAGGTGTA
B_23	TTCTGACCTGAAAGCGTAAGAATACGTGGCACAGACAATATTTTTGAATGGCTATT
B_24	AAGAAACAATGAAATAGCAATAGCTATCTTACCGAAGCCCTTTTTAAGAAAAGTAA
B_25	ATTGAGGAAGGTTATCTAAAATATCTTTAGGAGCACTAACAACTAATAGATTAGAG
B_26	ACAGGCAAGGCAAAGAATTAGCAAAATTAAGCAATAAAGCCTCAGAGCATAAAGCT
B_27	CTACGTTAATAAAACGAACTAACGGAACAACATTATTACAGGTAGAAAGATTCATC
B_28	TTTTATAATCAGTGAGGCCACCGAGTAAAAGAGTCTGTCCATCACGCAAATTAACC
B_29	AAAGAGGCAAAAGAATACACTAAAACACTCATCTTTGACCCCCAGCGATTATACCA
B_30	GTGCGGGCCTCTTCGCTATTACGCCAGCTGGCGAAAGGGGGATGTGCTGCAAGGCG

Supplementary Table 2: Byproduct sequences in cDNA library prepared with TSO.

Byproduct name	Sequence (5′–3′)
TSO	AAGCAGTGGTATCAACGCAGAGTACGGG
Complementary of TSO	CCCGTACTCTGCGTTGATACCACTGCTT
T30	TTTTTTTTTTTTTTTTTTTTTTTTT
A30	AAAAAAAAAAAAAAAAAAAAAAAAA
(AT)15	ATATATATATATATATATATATATATATAT
