# Supplementary material for: Development of a sequencing system for spatial decoding of DNA barcode molecules at single-molecule resolution
Source: Commun Biol. 2020 Dec 18;3:788. doi: 10.1038/s42003-020-01499-8 (PMC7749132; doi:10.1038/s42003-020-01499-8)
Supplement: Supplementary file 3 — Description of Additional Supplementary Files [file 42003_2020_1499_MOESM3_ESM.pdf]

Description of Additional Supplementary Files

File Name: Supplementary Data 1
Description: Source data underlying the plots shown in the main figures
