# Supplementary material for: Development of a sequencing system for spatial decoding of DNA barcode molecules at single-molecule resolution
Source: Commun Biol. 2020 Dec 18;3:788. doi: 10.1038/s42003-020-01499-8 (PMC7749132; doi:10.1038/s42003-020-01499-8)
Supplement: Supplementary file 5 — Reporting Summary [file 42003_2020_1499_MOESM5_ESM.pdf]

nature research

Corresponding author(s): Yusuke Oguchi
Sotaro Uemura

Last updated by author(s): Oct 12, 2020

Reporting Summary

Nature Research wishes to improve the reproducibility of the work that we publish. This form provides structure for consistency and transparency in reporting. For further information on Nature Research policies, see our Editorial Policies and the Editorial Policy Checklist.

Statistics

For all statistical analyses, confirm that the following items are present in the figure legend, table legend, main text, or Methods section.

n/a	Confirmed	
☐	☒	The exact sample size (*n*) for each experimental group/condition, given as a discrete number and unit of measurement
☐	☒	A statement on whether measurements were taken from distinct samples or whether the same sample was measured repeatedly
☐	☒	The statistical test(s) used AND whether they are one- or two-sided<br>*Only common tests should be described solely by name; describe more complex techniques in the Methods section.*
☒	☐	A description of all covariates tested
☒	☐	A description of any assumptions or corrections, such as tests of normality and adjustment for multiple comparisons
☐	☒	A full description of the statistical parameters including central tendency (e.g. means) or other basic estimates (e.g. regression coefficient) AND variation (e.g. standard deviation) or associated estimates of uncertainty (e.g. confidence intervals)
☐	☒	For null hypothesis testing, the test statistic (e.g. *F*, *t*, *r*) with confidence intervals, effect sizes, degrees of freedom and *P* value noted<br>*Give P values as exact values whenever suitable.*
☒	☐	For Bayesian analysis, information on the choice of priors and Markov chain Monte Carlo settings
☒	☐	For hierarchical and complex designs, identification of the appropriate level for tests and full reporting of outcomes
☒	☐	Estimates of effect sizes (e.g. Cohen's *d*, Pearson's *r*), indicating how they were calculated

Our web collection on statistics for biologists contains articles on many of the points above.

Software and code

Policy information about availability of computer code

Data collection	The custom code regulating our sequencing system is not freely available; the code is integrated with a commercially available software (Hamamatsu photonics) and works only with the system described in the methods section.
Data analysis	R(v3.5.1), STAR(v2.5.1b), RSEM(v1.3.0), HTseq-count(v0.11.2), BLAST(2.2.30+)

For manuscripts utilizing custom algorithms or software that are central to the research but not yet described in published literature, software must be made available to editors and reviewers. We strongly encourage code deposition in a community repository (e.g. GitHub). See the Nature Research guidelines for submitting code & software for further information.

Data

Policy information about availability of data

All manuscripts must include a data availability statement. This statement should provide the following information, where applicable:
- Accession codes, unique identifiers, or web links for publicly available datasets
- A list of figures that have associated raw data
- A description of any restrictions on data availability

The sequencing data from this study have been deposited in the DDBJ DRA database under the accession number DRA009022. The raw data referring to the plots shown in the main figures are provided in Supplementary Data 1.

Field-specific reporting

Please select the one below that is the best fit for your research. If you are not sure, read the appropriate sections before making your selection.

- [x] Life sciences
- [ ] Behavioural & social sciences
- [ ] Ecological, evolutionary & environmental sciences

For a reference copy of the document with all sections, see nature.com/documents/nr-reporting-summary-flat.pdf

Life sciences study design

All studies must disclose on these points even when the disclosure is negative.

Sample size	No calculation was performed.
Data exclusions	No data were excluded from the analyses.
Replication	All experiments were repeated at least twice except for that shown in Fig.2 and Fig.5
Randomization	Randomization was not applicable to this study.
Blinding	Blinding was not applicable to this study.

Reporting for specific materials, systems and methods

We require information from authors about some types of materials, experimental systems and methods used in many studies. Here, indicate whether each material, system or method listed is relevant to your study. If you are not sure if a list item applies to your research, read the appropriate section before selecting a response.

Materials & experimental systems

n/a	Involved in the study	
☐	☒	Antibodies
☐	☒	Eukaryotic cell lines
☒	☐	Palaeontology and archaeology
☒	☐	Animals and other organisms
☒	☐	Human research participants
☒	☐	Clinical data
☒	☐	Dual use research of concern

Methods

n/a	Involved in the study	
☒	☐	ChIP-seq
☒	☐	Flow cytometry
☒	☐	MRI-based neuroimaging

Antibodies

Antibodies used	TotalSeq™-A0383 anti-human CD55 Antibody(Biolegend), TotalSeq™-A0361 anti-human CD59 Antibody(Biolegend)
Validation	Validated by manufacturer.

Eukaryotic cell lines

Policy information about cell lines

Cell line source(s)	K562 cells (human lymphoblast, chronic myelogenous leukemia) was provided by the RIKEN BRC.
Authentication	The cell line was authenticated by the supplier.
Mycoplasma contamination	The cell line was tested and no contamination of mycoplasma.
Commonly misidentified lines (See ICLAC register)	No cell line is listed by ICLAC.
